# STL3/433: IATROTEK On-line: The Hellenic medical literature retrieval system

**DOI:** 10.2196/jmir.1.suppl1.e107

**Published:** 1999-09-19

**Authors:** PA Kokkinides, S Kolokithas, AE Germenis

**Affiliations:** ^1^The Athens Medical Society, AthensGreece

**Keywords:** Information Storage and Retrieval, Databases, Bibliographic, Online Systems

## Abstract

**Introduction:**

IATROTEK is a bilingual (Greek, English) database containing the references of about 15.000 articles (abstracts included), which have been published in 75 Greek biomedical journals since 1982. Until recently, the input in this database was performed by manual typing and the retrieval was available to the users only off line. Input automation was started before six months and on-line use of the database was officially inaugurated on May 8th, 1999 (www.mednet.gr/iatrotek).

**Methods:**

The on-line version of IATROTEK is implemented on a Linux-based server running PostgreSQL database server and Apache web server. All the software used is freeware.The user interface is comprised of several HTML forms, which are accessible by any WWW browser, and it is supported by certain CGI (Common Gateway Interface) scripts written in Perl. Searching can be performed providing single terms or terms combined by boolean operators, on the following fields: title, authors, key-words, abstract in both Greek and English. Matching records include the title and reference of the articles as well as links to the Greek and the English abstracts. Links providing further searching on authors' names and key-words are available on the page of abstracts. Concerning the input in the database, it is performed by the use of appropriate filters (format transformers) on the data provided by the publishers. Under construction is the automation of key-word assigning procedure and a stemming algorithm for Greek words.

**Results:**

During the first fifteen days of its on-line operation, the database is queried with a mean rate of 48 requests/day (20% of them from abroad). Bearing in mind the small number of Greek doctors having access to the Web (2,500-3,000 out of 35,000), this is a fact signifying their warm interest on the on-line use of the IATROTEK.

**Discussion:**

Compared to the availability of the international medical literature through the Web, the literature written in national languages is not equally favored by the Internet explosion. Efforts, like that of IATROTEK on-line, towards the dissemination of locally produced medical information through the Web will overcome the serious obstacles confronted by the national journals.

